# Effect of Sodium Thiosulfate Pre-Treatment on Renal Ischemia-Reperfusion Injury in Kidney Transplantation

**DOI:** 10.3390/ijms25179529

**Published:** 2024-09-02

**Authors:** Pierce Nelson, George J. Dugbartey, Liam McFarlane, Patrick McLeod, Sally Major, Jifu Jiang, Caroline O’Neil, Aaron Haig, Alp Sener

**Affiliations:** 1 Department of Microbiology & Immunology, Schulich School of Medicine and Dentistry, Western University, London, ON N6A 5C1, Canada; 2 Matthew Mailing Center for Translational Transplant Studies, London Health Sciences Center, Western University, London, ON N6A 5A5, Canada; gdugbart@uwo.ca (G.J.D.);; 3 Multi-Organ Transplant Program, London Health Sciences Center, Western University, London, ON N6A 5A5, Canada; 4 London Health Sciences Center, Department of Surgery, Western University, London, ON N6A 5A5, Canada; 5 Department of Pharmacology & Toxicology, School of Pharmacy, College of Health Sciences, University of Ghana, Accra P.O. Box LG43, Ghana; 6 The Molecular Pathology Core, Robarts Research Institute, London, ON N6A 5A5, Canada; 7 Department of Pathology and Laboratory Medicine, Western University, London, ON N6A 5A5, Canada

**Keywords:** kidney transplantation, ischemia-reperfusion injury (IRI), sodium thiosulfate (STS), necroptosis, University of Wisconsin (UW) solution, static cold storage (SCS)

## Abstract

We recently reported in a rat model of kidney transplantation that the addition of sodium thiosulfate (STS) to organ preservation solution improved renal graft quality and prolonged recipient survival. The present study investigates whether STS pre-treatment would produce a similar effect. In vitro, rat kidney epithelial cells were treated with 150 μM STS before and/or during exposure to hypoxia followed by reoxygenation. In vivo, donor rats were treated with PBS or 2.4 mg/kg STS 30 min before donor kidneys were procured and stored in UW or UW+150 μM STS solution at 4 °C for 24 h. Renal grafts were then transplanted into bilaterally nephrectomised recipient rats which were then sacrificed on post-operative day 3. STS pre-treatment significantly reduced cell death compared to untreated and other treated cells in vitro (*p* < 0.05), which corresponded with our in vivo result (*p* < 0.05). However, no significant differences were observed in other parameters of tissue injury. Our results suggest that STS pre-treatment may improve renal graft function after transplantation.

## 1. Introduction

End-stage renal disease (ESRD) is defined as kidney failure requiring renal replacement therapy (RRT) to sustain life, with dialysis and kidney transplantation being the current forms of RRT [1]. In 2010, it was estimated that at least 2.6 million people worldwide were receiving RRT, with this number expected to double to over 5.4 million people in 2030 [1]. This increase is concerning considering that over 1.2 million people die each year due to chronic kidney disease [2]. Kidney transplantation is the treatment of choice when compared to dialysis due to improved survival rates, reduced costs, and higher quality of life [3,4,5]. However, the success of this life-saving intervention is hampered by ischemia-reperfusion injury (IRI), an injury caused by the cessation (ischemia) and subseuent restoration (reperfusion) of blood flow to an organ [6,7,8]. Kidney transplantation inherently results in IRI since the donor kidney becomes ischemic upon removal from the donor and undergoes reperfusion upon implantation into the recipient [8]. In renal IRI, overproduction of reactive oxygen species (ROS; destructive mediators of cell and tissue injury) during reperfusion leads to downstream apoptosis, necrosis, and inflammation, which collectively lead to impaired graft function and other post-transplant complications [6,9,10,11,12,13,14,15,16]. Necroptosis and ferroptosis are the major forms of regulated necrosis while macrophages and neutrophils are the primary mediators of inflammation in renal IRI [7,17,18,19].

The current gold standard for preserving donor kidneys and mitigating IRI during transplantation is static cold storage (SCS) in University of Wisconsin (UW) preservation solution on ice at 4 °C [20]. Although SCS attempts to mitigate IRI, prolonged cold storage contributes to IRI and increased incidence of delayed graft function [8,21]. As a result, much research has focused on the modification of preservation solutions with additives and pharmacological supplements to further inhibit IRI [8]. Hydrogen sulfide (H_2_S), the third established member of a family of gasotransmitters, is one such pharmacological agent that has gained scientific attention in protecting against renal IRI [22]. As a gasotransmitter, H_2_S is produced endogenously in mammalian cells via the enzymatic actions of cystathionine-β-synthase (CBS), cystathionine-γ-lyase (CSE), and 3-mercaptopyruvate sulfurtransferase (MPST) [23,24,25,26]. Although several compounds have been identified as H_2_S donors, only sodium thiosulfate (STS) is currently clinically viable since it has already been approved by both the US Food and Drug Administration and Health Canada for the treatment of acute cyanide poisoning [27,28]. We recently showed, in a rat model of kidney transplantation, that supplementation of UW solution with STS significantly attenuated cold renal IRI and improved donor kidney function, leading to prolonged recipient survival [29]. However, much of the previous literature on using H_2_S donors to protect against IRI has focused on administering these molecules either during the ischemic phase or following reperfusion. Pre-treatment of the kidney transplant donor with an H_2_S donor before the renal graft is removed and exposed to ischemia may also confer protection. Previous studies have shown that H_2_S pre-treatment is protective in animal models of cold IRI induced by lung transplantation [30,31,32]. 

Currently, it is unknown whether the administration of H_2_S to kidney donors before organ procurement protects donor kidneys against IRI induced by kidney transplantation. Therefore, the aim of this study was to determine if the administration of STS to donor rats before organ procurement protects the donor kidneys against transplantation-induced IRI and elucidate the mechanisms of this protection. We hypothesized that STS treatment before exposure to ischemia would protect kidney grafts against transplantation-induced IRI by suppressing oxidative stress, cell death, and inflammation, leading to improved graft function and recipient survival.

## 2. Results

### 2.1. Effect of STS Pre-Treatment on Rat Renal Epithelial Cell Viability in an In Vitro Model of Hypoxia–Reoxygenation Injury

**Figure 1:**
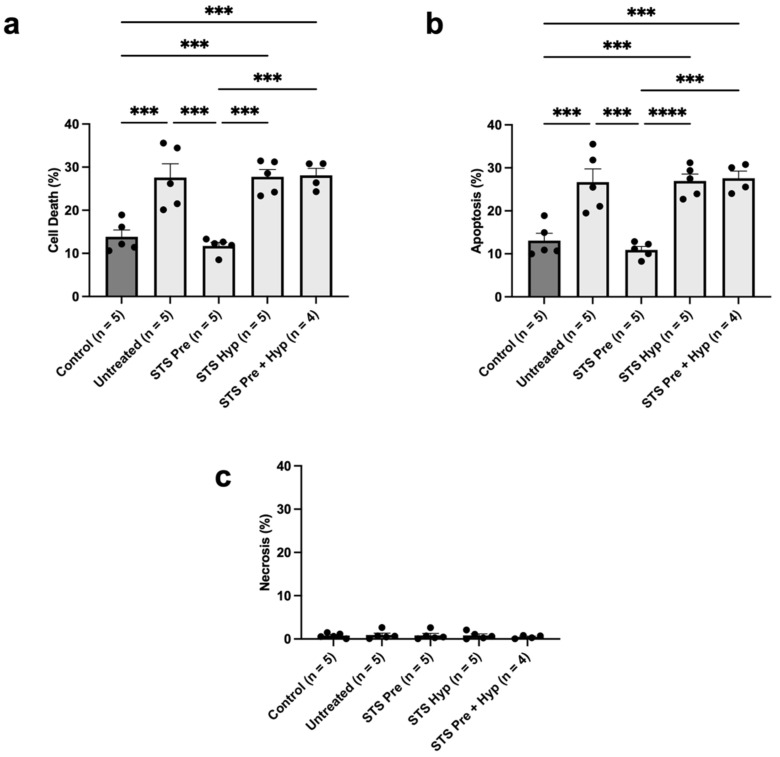
Effect of STS pre-treatment on cell death in an in vitro model of hypoxia–reoxygenation injury. Rat kidney epithelial (NRK-52E) cells were exposed to 24 h of 37 °C hypoxia in serum-free media followed by reoxygenation in serum-positive media for 24 h at 37 °C. Control cells were cultured in serum-positive media under normoxic conditions for the same period. Cells were stained with FITC-Annexin V (apoptosis) and propidium iodide (necrosis) for analysis via flow cytometry. Experimental cells received no treatment or were treated with 150 μM STS for 2 h before hypoxia (STS Pre), during hypoxia (STS Hyp), or both before and during hypoxia (STS Pre + Hyp). (**a**) Mean cell death percentage (FITC-Annexin^+^ and/or PI^+^). (**b**) Mean apoptosis percentage (FITC-Annexin^+^/PI^−^). (**c**) Mean necrosis percentage (FITC-Annexin V^−^/PI^+^). Bars represent mean ± SEM (n = 4). Means were compared using one-way ANOVA and Tukey’s post hoc test. *** *p* < 0.001, **** *p* < 0.0001. STS: sodium thiosulfate.

To first determine whether STS pre-treatment has the potential to protect against renal IRI, we used an in vitro model of hypoxia–reoxygenation injury. In this model, NRK-52E cells were exposed to 37 °C hypoxia and reoxygenation followed by cell viability analysis via flow cytometry. Cells received no treatment or were treated with 150 μM STS for two hours before hypoxia, during hypoxia, or both before and during hypoxia via the addition of STS to the cell culture medium. STS pre-treatment significantly reduced NRK-52E cell death following reoxygenation compared to the untreated control group and all other treatment groups (Figure 1a; *p* < 0.05). However, cell death in the STS pre-treatment combined with the hypoxic treatment group was not significantly different from the untreated group (*p* > 0.05) but was significantly higher than the normoxia control group (*p* < 0.05). Similarly, STS pre-treatment substantially reduced apoptosis compared to the untreated group and other treatment groups (Figure 1b; *p* < 0.05). However, apoptosis levels in the STS pre-treatment combined with the hypoxic treatment group were not significantly different compared to the untreated group (*p* > 0.05) but significantly higher than the normoxia control group (*p* < 0.05). There was no significant difference found in necrosis levels between any of the groups (Figure 1c). Notably, necrosis levels were relatively lower compared to apoptosis levels for all groups. In summary, STS pre-treatment alone attenuated cell death, specifically apoptosis, in our in vitro model of hypoxia–reoxygenation injury, whereas STS treatment during hypoxia alone or combined with pre-treatment did not confer protection.

### 2.2. Effect of STS Pre-Treatment on Transplant Recipient Survival and Renal Function

**Figure 2:**
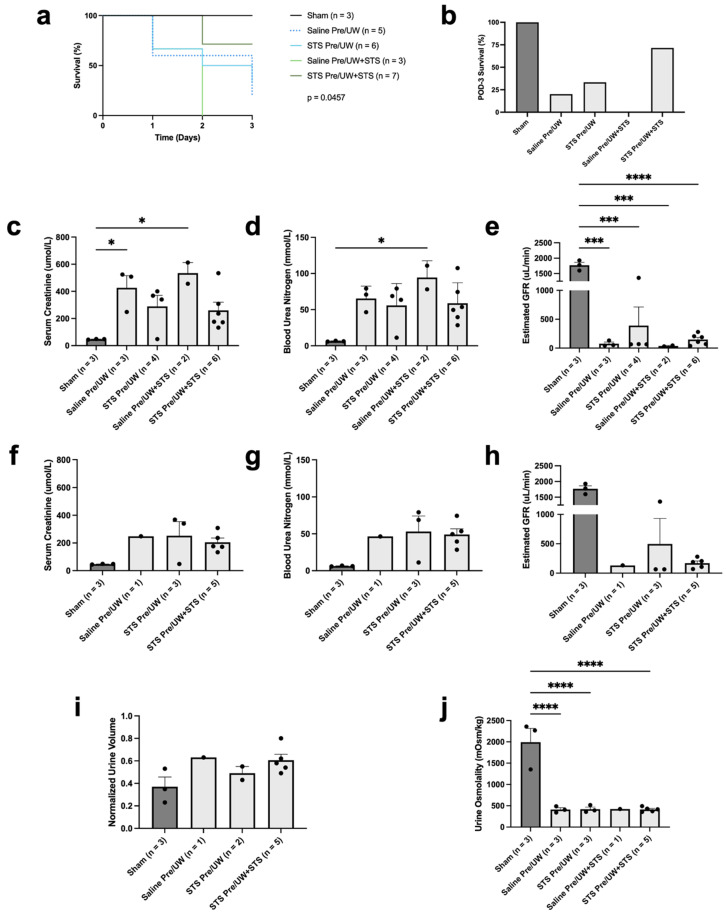
Effect of STS pre-treatment on survival and renal function in recipient rats following kidney transplantation. Donor rats received an injection of PBS (Saline Pre) or 2.4 mg STS/kg of body weight (STS Pre) intravenously 30 min before donor kidney procurement. Donor kidneys were stored for 24 h at 4 °C in either UW or UW + 150 μM STS (UW+STS). Sham rats underwent a midline incision only. (**a**) Kaplan–Meier survival curves of survival data. Survival curves were compared using a log-rank test and the corresponding p-value is indicated. (**b**) POD-3 survival rate of the different experimental groups. (**c**–**e**) Terminal serum creatinine, BUN, and eGFR. (**f**–**h**) POD-3 serum creatinine, BUN, and eGFR. (**i**) urine volume from POD-2 to 3 normalized to water intake. (**j**) Urine osmolality from POD-2 to 3 urine samples. Bars represent mean ± SEM. Means were compared using one-way ANOVA and Tukey’s post hoc test. * *p* < 0.05, *** *p* < 0.001, **** *p* < 0.0001. POD: post-operative day; BUN: blood urea nitrogen; eGFR: estimated glomerular filtration rate; UW: University of Wisconsin solution; STS: sodium thiosulfate.

To assess the protective effects of STS pre-treatment in vivo, we performed syngeneic kidney transplantation in rats where donor rats were pre-treated with 2.4 mg STS/kg of body weight or PBS 30 min before donor kidney removal. The procured renal grafts were stored for 24 h in UW or UW solution supplemented with 150 μM STS. Sham rats did not receive kidney transplants and underwent a midline incision only. Following transplantation or midline incision (Sham), rats were monitored until POD-3. The number of rats that survived (not found dead or preemptively sacrificed based on a standardized animal welfare scoring algorithm) to POD-3 was used to calculate POD-3 survival rates for each group. A description of the animal groups used in this study is shown in Table 1. As expected, survival of the Sham group was highest (POD-3 survival = 100.00%), followed by STS pre-treatment combined with UW+STS (POD-3 survival = 71.43%), STS pre-treatment alone (POD-3 survival = 33.33%), control (POD-3 survival = 20.00%), and UW+STS-only (POD-3 survival = 0.00%) (Figure 2a,b). A log-rank test indicated there was a significant difference between the survival curves (Figure 2a; *p* < 0.05). However, post-hoc analysis using the Holm–Bonferroni method revealed no significant differences in any of the treatment groups compared to the control group. Similarly, there were no significant differences in any of the survival curves compared to Sham.

To determine whether STS pre-treatment improved renal graft function, we assessed serum creatinine and BUN in recipient rats at the time of sacrifice. We found no significant differences in terminal serum creatinine levels in any of the treatment groups compared to the control group (Figure 2c). However, serum creatinine levels in the control and UW+STS-only groups were significantly higher compared to the Sham group (*p* < 0.05), whereas there were no significant differences between the two pre-treatment groups and the Sham group. There were also no significant differences in terminal BUN levels in any of the treatment groups compared to the control group (Figure 2d). However, BUN levels in the UW+STS-only group were significantly higher compared to the Sham group (Figure 2d; *p* < 0.05). In addition, there were no significant differences in terminal eGFR in any of the treatment groups compared to the control group (Figure 2e). As expected, eGFR in the Sham group was significantly higher compared to all the treatment groups (*p* < 0.05). Since blood markers of renal function can vary with respect to time post-transplantation, we also examined these markers exclusively in recipient rats that survived to POD-3. No statistical analysis was performed because we only had blood data for one recipient rat in the control group. STS pre-treatment combined with UW+STS showed a marginal reduction in serum creatinine (Figure 2f) and improvement in eGFR (Figure 2h) but did not affect BUN levels (Figure 2g) on POD-3.

To further examine the effect of STS pre-treatment on renal graft function, we also assessed urine output and osmolality from POD-2 to POD-3. Transplantation led to upward trends in normalized urine volume output from POD-2 to 3 across all experimental groups when compared to Sham-operated rats (Figure 2i). No statistical analysis was performed since there was only one rat in the control group that survived to POD-3. We also found no significant differences in urine osmolality between any of the treatment groups compared to the control group (Figure 2j). However, urine osmolality was significantly lower in all experimental groups compared in to the Sham group (*p* < 0.05), except for in the UW+STS-only group, which was not included in statistical analysis due to the sample size being n = 1. Collectively, STS pre-treatment did not significantly affect recipient survival and renal graft function.

### 2.3. Effect of STS Pre-Treatment on Oxidative Stress in Renal Grafts

**Figure 3:**
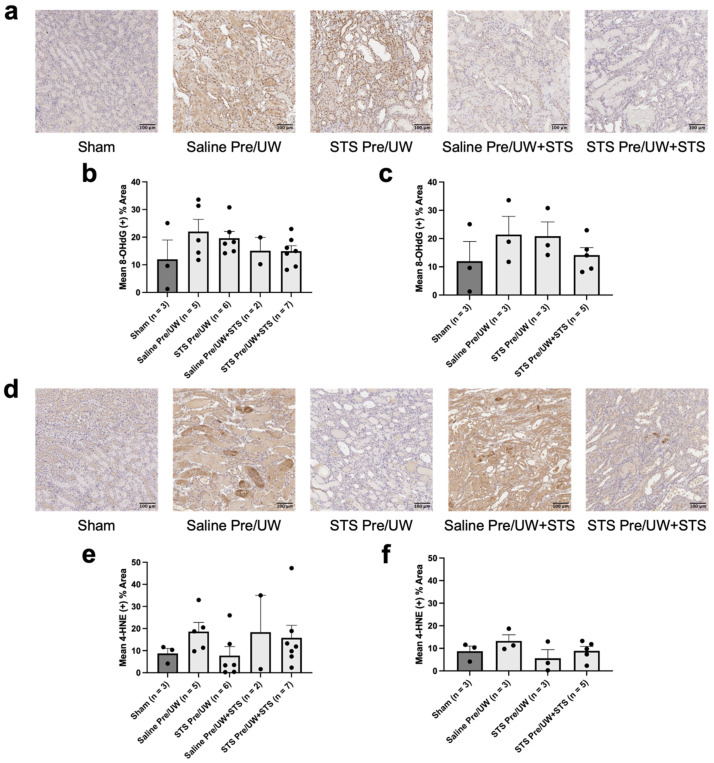
Effect of STS pre-treatment on tissue oxidative stress in renal grafts following transplantation. Donor rats received an injection of PBS (Saline Pre) or 2.4 mg STS/kg of body weight (STS Pre) 30 min before kidney procurement. Kidneys were stored for 24 h at 4 °C in either UW or UW + 150 μM STS (UW+STS). Sham rats underwent a midline incision only. (**a**) Representative images of kidney sections stained with 8-OHdG. (**b**) Terminal and (**c**) POD-3 mean positive 8-OHdG staining. (**d**) Representative images of kidney sections stained with 4-HNE. (**e**) Terminal and (**f**) POD-3 mean positive 4-HNE staining. Scale bars are 100 μm. Bars represent mean ± SEM. Means were compared using one-way ANOVA and Tukey’s post hoc test. POD: post-operative day; UW: University of Wisconsin solution; 8-OHdG: 8-hydroxy-2′-deoxyguanosine; 4-HNE: 4-Hydroxynonenal; STS: sodium thiosulfate.

To assess potential molecular mechanisms of action of STS pre-treatment, we conducted histological and qPCR analyses on renal grafts procured from the same Sham and transplant recipient rats used in our survival and renal graft function analyses. First, to determine whether STS pre-treatment suppressed oxidative stress, we performed IHC staining targeting 8-OHdG (oxidative DNA damage marker) and 4-HNE (lipid peroxidation marker) (Figure 3a). We found there were no significant differences in terminal oxidative DNA damage and lipid peroxidation levels in renal grafts following transplantation in any of the treatment groups compared to the control group (Figure 3b,e). Also, terminal oxidative DNA damage and lipid peroxidation levels were not significantly different in the Sham group compared to the control group.

We then compared 8-OHdG and 4-HNE levels exclusively in rats that were either sacrificed or found dead on POD-3 to enable comparisons at a more similar time point post-transplantation (Figure 3c,f). Similarly, we found no significant differences in POD-3 oxidative DNA damage and lipid peroxidation levels in renal grafts following transplantation in any of the treatment groups compared to the control group. Additionally, POD-3 oxidative DNA damage and lipid peroxidation levels were not significantly different in the Sham group compared to the control group.

**Figure 4:**
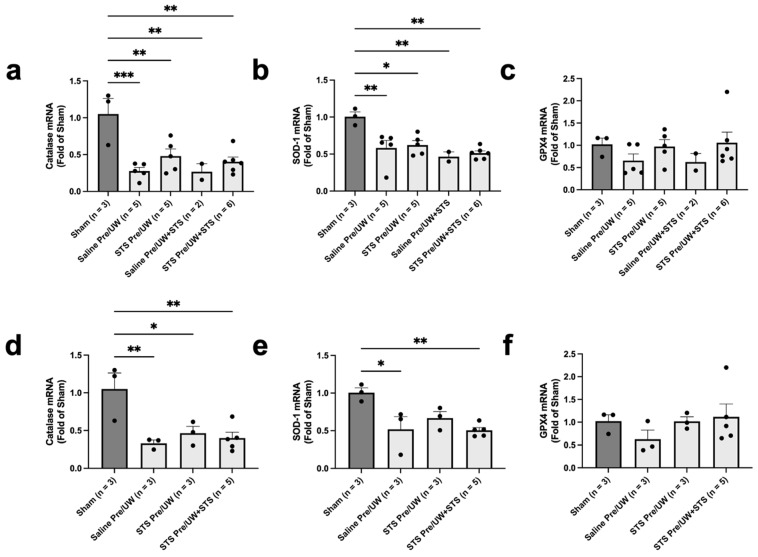
Effect of STS pre-treatment on antioxidant gene expression in renal grafts following kidney transplantation. Donor rats received an injection of PBS (Saline Pre) or 2.4 mg STS/kg of body weight (STS Pre) 30 min before kidney procurement. Kidneys were stored for 24 h at 4 °C in either UW or UW + 150 μM STS (UW+STS). Sham rats underwent a midline incision only. Total RNA was extracted from renal tissue and reverse transcribed to cDNA for qPCR analysis targeting catalase, SOD-1, and GPX4. (**a**–**c**) Terminal and (**d**–**f**) POD-3 mRNA expression levels. Bars represent mean ± SEM. Means were compared using one-way ANOVA and Tukey’s post hoc test. * *p* < 0.05, ** *p* < 0.01, *** *p* < 0.001. POD: post-operative day; UW: University of Wisconsin solution; SOD-1: superoxide dismutase 1; GPX4: glutathione peroxidase 4; STS: sodium thiosulfate.

To determine whether STS pre-treatment mediated changes in antioxidant gene levels, we performed qPCR in renal tissue to assess the mRNA expression of catalase, SOD-1, and GPX4. We found no significant differences in terminal catalase and SOD-1 mRNA expression in any of the treatment groups compared to the control group (Figure 4a,b). However, terminal catalase and SOD-1 mRNA expression were significantly reduced in all experimental groups compared to Sham (*p* < 0.05). We found no significant differences in terminal GPX4 expression in any of the treatment groups compared to the control group (Figure 4c). There was also no significant difference in Sham GPX4 expression compared to the control group.

When focusing on mRNA expression exclusively in rats sacrificed or found dead on POD-3, we found no significant differences in POD-3 catalase and SOD-1 mRNA levels in any of the treatment groups compared to the control group (Figure 4d,e). However, catalase mRNA expression was significantly elevated in the Sham group compared to all experimental groups (Figure 4d; *p* < 0.05). Additionally, SOD-1 mRNA expression on POD-3 was significantly higher in the Sham group compared to the control and STS pre-treatment combined with UW+STS groups (Figure 4e; *p* < 0.05). However, SOD-1 expression in the STS pre-treatment alone group was not significantly different compared to Sham. Similarly, there were no significant differences in POD-3 GPX4 expression in any of the treatment groups compared to the control group (Figure 4f). GPX4 expression on POD-3 in the Sham group was also not significantly different compared to the control group. Altogether, STS pre-treatment did not significantly modulate oxidative stress levels in renal grafts following kidney transplantation.

### 2.4. Effect of STS Pre-Treatment on Cell Death in Renal Grafts

**Figure 5:**
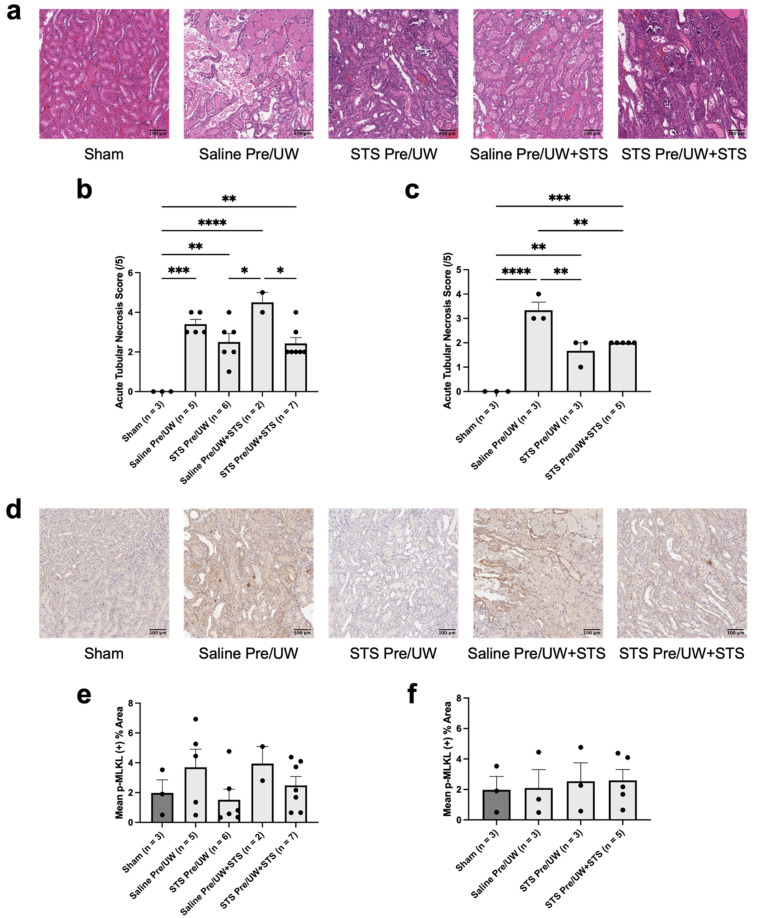
Effect of STS pre-treatment on necrosis in renal grafts following kidney transplantation. Donor rats received an injection of PBS (Saline Pre) or 2.4 mg STS/kg of body weight (STS Pre) 30 min before kidney procurement. Kidneys were stored for 24 h at 4 °C in either UW or UW + 150 μM STS (UW+STS). Sham rats underwent a midline incision only. (**a**) Representative images of kidney sections stained with H&E. (**b**) Terminal and (**c**) POD-3 mean ATN scores assessed by a blinded renal pathologist. (**d**) Representative images of kidney sections stained with p-MLKL. (**e**) Terminal and (**f**) POD-3 mean positive p-MLKL staining. Scale bars are 100 μm. Bars represent mean ± SEM. Means were compared using one-way ANOVA and Tukey’s post hoc test. * *p* < 0.05, ** *p* < 0.01, *** *p* < 0.001, **** *p* < 0.0001. POD: post-operative day; UW: University of Wisconsin solution; ATN: acute tubular necrosis; p-MLKL: phospho-mixed lineage kinase domain-like protein; STS: sodium thiosulfate.

To determine the amount of necrosis present in renal grafts following transplantation, we stained renal tissue sections with H&E for scoring by a blinded renal pathologist (Figure 5a–c). Terminal acute tubular necrosis (ATN) scores in all the treatment groups were not significantly different compared to the control group (Figure 5b). However, both pre-treatment groups had significantly lower ATN scores compared to the UW+STS-only group (*p* < 0.05). All experimental groups had significantly higher terminal ATN scores compared to Sham (*p* < 0.05). We compared ATN scores exclusively in rats that were either sacrificed or found dead on POD-3. Both STS pre-treatment and STS pre-treatment combined with UW+STS had significantly lower POD-3 ATN scores compared to the control group (Figure 5c; *p* < 0.05). ATN scores on POD-3 were significantly higher for all experimental groups compared to the Sham group (*p* < 0.05).

To determine whether STS pre-treatment specifically reduced necroptosis, a form of regulated necrosis, we performed IHC staining targeting p-MLKL (a marker of necroptosis) (Figure 5d–f). We found no significant differences in terminal necroptosis levels in the Sham, STS pre-treatment alone, UW+STS alone, and STS pre-treatment combined with UW+STS groups compared to the control group (Figure 5e). Similarly, when examining necroptosis levels in rats that were sacrificed or found dead on POD-3, no significant differences in necroptosis levels were found in the Sham, STS pre-treatment alone, and STS pre-treatment combined with UW+STS groups compared to the control group (Figure 5f). Taken altogether, STS pre-treatment reduced ATN but not necroptosis in renal grafts post-transplantation.

**Figure 6:**
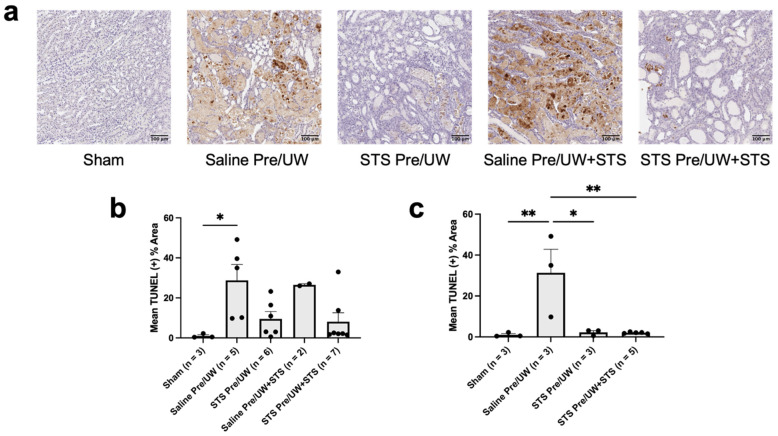
Effect of STS pre-treatment on apoptosis in renal grafts following kidney transplantation. Donor rats received an injection of PBS (Saline Pre) or 2.4 mg STS/kg of body weight (STS Pre) 30 min before kidney procurement. Kidneys were stored for 24 h at 4 °C in either UW or UW + 150 μM STS (UW+STS). Sham rats underwent a midline incision only. (**a**) Representative images of kidney sections stained with TUNEL. Scale bars are 100 μm. (**b**) Terminal and (**c**) POD-3 mean positive TUNEL staining. Bars represent mean ± SEM. Means were compared using one-way ANOVA and Tukey’s post hoc test. * *p* < 0.05, ** *p* < 0.01. POD: post-operative day; UW: University of Wisconsin solution; TUNEL: terminal deoxynucleotidyl transferase dUTP nick end labeling; STS: sodium thiosulfate.

To determine the amount of apoptosis present in renal grafts following transplantation, we stained renal tissue sections with TUNEL (Figure 6a). We found that STS pre-treatment alone and UW+STS alone terminal apoptosis levels were not significantly different compared to the control group (Figure 6b). Although not statistically significant, the difference in apoptosis levels between the control and STS pre-treatment combined with UW+STS groups approached statistical significance (*p* = 0.05). Terminal apoptosis was significantly higher in the control group compared to the Sham group (*p* < 0.05). However, apoptosis levels in all the treatment groups were not significantly different compared to those in the Sham group.

We then compared TUNEL staining exclusively in rats that were either sacrificed or found dead on POD-3 (Figure 6c). We found that POD-3 apoptosis was significantly lower in both the STS pre-treatment alone and STS pre-treatment combined with UW+STS groups compared to the control group (*p* < 0.05). Similarly, apoptosis in the Sham group was significantly lower compared to the control group (*p* < 0.05). However, apoptosis levels in both pre-treatment groups were not significantly different compared to Sham. Collectively, STS pre-treatment reduced apoptosis levels in renal grafts following transplantation.

### 2.5. Effect of STS Pre-Treatment on Inflammation and Injury in Renal Grafts

**Figure 7:**
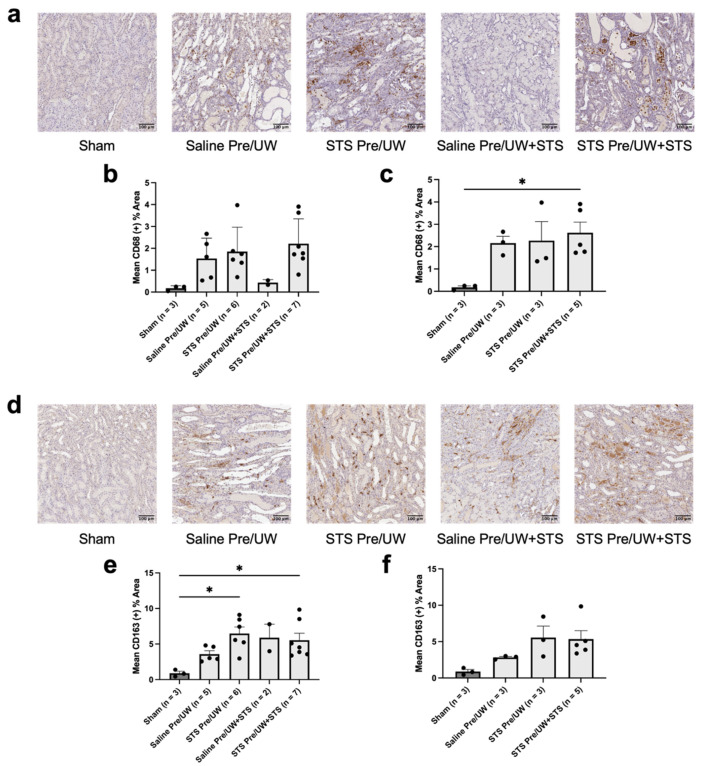
Effect of STS pre-treatment on macrophage infiltration in renal grafts following kidney transplantation. Donor rats received an injection of PBS (Saline Pre) or 2.4 mg STS/kg of body weight (STS Pre) 30 min before kidney procurement. Kidneys were stored for 24 h at 4 °C in either UW or UW + 150 μM STS (UW+STS). Sham rats underwent a midline incision only. (**a**) Representative images of kidney sections stained with CD68. (**b**) Terminal and (**c**) POD-3 mean positive CD68 staining. (**d**) Representative images of kidney sections stained with CD163. (**e**) Terminal and (**f**) POD-3 mean positive CD163 staining. Scale bars are 100 μm. Bars represent mean ± SEM. Means were compared using one-way ANOVA and Tukey’s post hoc test. * *p* < 0.05. POD: post-operative day; UW: University of Wisconsin solution; STS: sodium thiosulfate.

To determine the effect of STS pre-treatment on inflammation induced by IRI, we performed IHC staining on renal grafts targeting CD68 (pan-macrophage marker) (Figure 7a–c). Although one-way ANOVA showed a significant difference amongst the groups (*p* < 0.05), no individual pairwise comparison was statistically significant (Figure 7b). Specifically, there were no significant differences in macrophage infiltration in any of the treatment groups compared to the control group. There were also no significant differences in macrophage infiltration in the control, UW+STS alone, and STS pre-treatment alone groups compared to Sham. However, the difference in macrophage infiltration in the STS pre-treatment combined with the UW+STS group compared to Sham approached statistical significance (*p* = 0.05). We subsequently compared CD68 staining exclusively in rats that were either sacrificed or found dead on POD-3 (Figure 7c). There were no significant differences in POD-3 macrophage infiltration in both pre-treatment groups compared to the control group. The control and STS pre-treatment alone groups did not have significantly different macrophage levels compared to Sham. However, POD-3 macrophage infiltration was significantly higher in the STS pre-treatment combined with the UW+STS group compared to Sham (*p* < 0.05).

To characterize the macrophage population present in the renal grafts, we performed IHC staining targeting CD163 (M2 macrophage marker) (Figure 7d–f). We found that STS pre-treatment alone, UW+STS alone, and STS pre-treatment combined with UW+STS terminal M2 macrophage levels were not significantly different compared to the control group (Figure 7e). Terminal M2 macrophage infiltration was significantly higher in the STS pre-treatment alone and STS pre-treatment combined with UW+STS groups compared to Sham (*p* < 0.05). In contrast, M2 macrophage infiltration levels in the control and UW+STS alone groups were not significantly different compared to Sham. We also assessed CD163 staining exclusively in rats that were sacrificed or found dead on POD-3 (Figure 7f). STS pre-treatment alone or combined with UW+STS did not have significantly different POD-3 M2 macrophage levels compared to the control group. No significant differences were found in POD-3 M2 macrophage levels in the control, STS pre-treatment alone, or STS pre-treatment combined with UW+STS groups compared to Sham.

**Figure 8:**
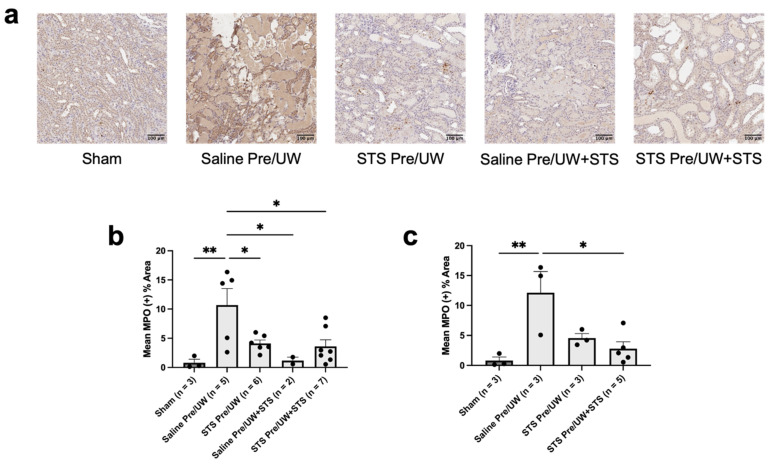
Effect of STS pre-treatment on neutrophil infiltration in renal grafts following kidney transplantation. Donor rats received an injection of PBS (Saline Pre) or 2.4 mg STS/kg of body weight (STS Pre) 30 min before kidney procurement. Kidneys were stored for 24 h at 4 °C in either UW or UW + 150 μM STS (UW+STS). Sham rats underwent a midline incision only. (**a**) Representative images of kidney sections stained with MPO. (**b**) Terminal and POD-3 (**c**) mean positive MPO staining. Scale bars are 100 μm. Bars represent mean ± SEM. Means were compared using one-way ANOVA and Tukey’s post hoc test. * *p* < 0.05, ** *p* < 0.01. POD: post-operative day; UW: University of Wisconsin solution; MPO: myeloperoxidase; STS: sodium thiosulfate.

We also performed IHC staining targeting MPO (a neutrophil marker) in the renal grafts to determine whether STS pre-treatment could inhibit neutrophil infiltration caused by IRI (Figure 8a). We found that STS pre-treatment alone, STS pre-treatment combined with UW+STS, and UW+STS alone significantly reduced terminal neutrophil infiltration in the renal grafts compared to the control group (Figure 8b; *p* < 0.05). Terminal neutrophil infiltration levels in these treatment groups were not significantly different compared to Sham, while neutrophil infiltration in the Sham group was significantly lower compared to the control group (*p* < 0.05). We found similar trends when assessing neutrophil infiltration only in rats that were sacrificed or found dead on POD-3 (Figure 8c). POD-3 neutrophil levels in both Sham and STS pre-treatment combined with UW+STS were not significantly different from each other but were significantly lower compared to the control group (*p* < 0.05). Neutrophil infiltration in the STS pre-treatment alone group was not significantly different from the control group but was also not significantly different from the Sham and STS pre-treatment combined with UW+STS groups. In summary, STS pre-treatment did not significantly affect macrophage levels and polarization but did reduce neutrophil infiltration in renal grafts following transplantation.

**Figure 9:**
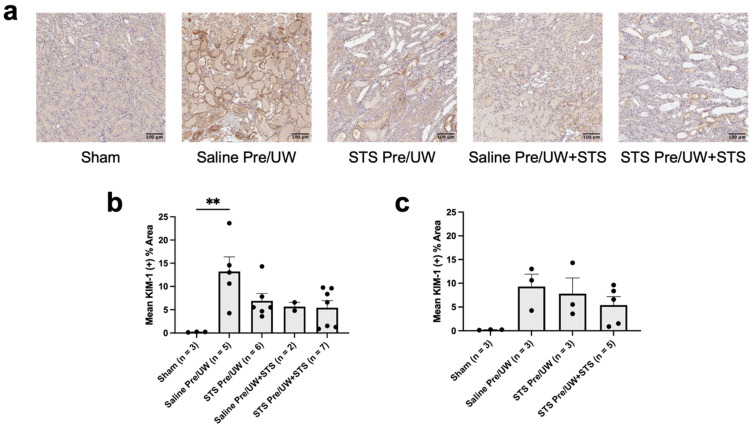
Effect of STS pre-treatment on KIM-1 expression in renal grafts following kidney transplantation. Donor rats received an injection of PBS (Saline Pre) or 2.4 mg STS/kg of body weight (STS Pre) 30 min before kidney procurement. Kidneys were stored for 24 h at 4 °C in either UW or UW + 150 μM STS (UW+STS). Sham rats underwent a midline incision only. (**a**) Representative images of kidney sections stained with KIM-1. (**b**) Terminal and (**c**) POD-3 mean positive KIM-1 staining. Scale bars are 100 μm. Bars represent mean ± SEM. Means were compared using one-way ANOVA and Tukey’s post hoc test. ** *p* < 0.01. POD: post-operative day; UW: University of Wisconsin solution; KIM-1: Kidney Injury Molecule-1; STS: sodium thiosulfate.

To assess the degree of tissue injury in the renal grafts following transplantation, we performed IHC staining targeting KIM-1 (kidney injury marker) (Figure 9a). We found that STS pre-treatment alone, STS pre-treatment combined with UW+STS, and UW+STS alone did not show a significant difference in terminal KIM-1 expression levels compared to the control group (Figure 9b). Terminal KIM-1 expression was also not significantly different between the Sham and treatment groups. However, terminal KIM-1 expression was significantly lower in the Sham group compared to the control group (*p* < 0.05). When analyzing KIM-1 expression only in rats that were sacrificed or found dead on POD-3, no significant differences were found in KIM-1 expression in any of the treatment groups compared to the control group (Figure 9c). Similarly, POD-3 KIM-1 expression was not significantly different in any of the experimental groups compared to the Sham group. Altogether, STS pre-treatment did not significantly reduce KIM-1 expression in renal grafts post-transplantation.

## 3. Discussion

In this study, we found that STS pre-treatment protected rat kidney epithelial cells against in vitro hypoxia–reoxygenation injury, and there was some evidence that STS pre-treatment may lead to improved graft outcomes and prolonged recipient survival in a rat model of syngeneic kidney transplantation.

In our in vitro model of hypoxia–reoxygenation injury, we found that rat kidney epithelial cells treated with STS for two hours before hypoxia had less cell death compared to untreated cells and similar cell death compared to healthy cells. STS pre-treatment mainly reduced apoptosis in this model, whereas necrosis was not affected. These findings align with a previous study in which treatment of human kidney epithelial cells with sodium hydrosulfide (NaHS), a non-clinically viable H_2_S donor, for 30 min before exposure to hypoxia–reoxygenation injury improved cell viability and reduced apoptosis [33]. The specific reduction of apoptosis found in our present study also aligns with previous studies showing that STS binds to the active site of caspase 3 and persulfidates, a cysteine residue, thereby inhibiting caspase 3 activity [34,35]. However, treatment of rat kidney epithelial cells during hypoxia rather than before hypoxia did not confer protection against cell death in the present study. In contrast, we previously found that STS treatment of rat kidney epithelial cells during hypoxia protected against hypoxia–reoxygenation injury [29]. These contradictory findings could be due to a difference in hypoxic temperatures, since we exposed cells to 37 °C hypoxia in the current study, while our previous study used 10 °C hypoxia. We have also previously shown that STS treatment during hypoxia at 21 °C in pig kidney epithelial cells and 4 °C and 10 °C in rat kidney epithelial cells is more cytoprotective than treatment during hypoxia at 37 °C [36,37]. Thus, the temperature at which hypoxia occurs likely plays a key role in the level of protection conferred by STS treatment administered during the hypoxic phase of our in vitro models. Surprisingly, STS pre-treatment combined with treatment during hypoxia also did not confer protection, suggesting that STS treatment during hypoxia abrogated the protection conferred by STS pre-treatment alone. This unexpected finding also contrasted with the findings of our in vivo model, where STS pre-treatment combined with supplementation to preservation solution showed some protection against renal IRI. A potential reason for this discrepancy could be transcriptomic and proteomic differences in immortalized renal proximal tubular epithelial cells, such as the NRK-52E cell line, compared to native renal proximal tubules. Khundmiri et al. showed that less than 50% of the transcriptome and proteome of the NRK-52E cell line overlapped with that of native renal proximal tubules [38]. Differences in mRNA and protein expression could theoretically lead to differences in cell survival responses toward STS treatment administered during hypoxia. Potential differences in survival responses in the NRK-52E cell line may explain the discrepancy observed in the combination treatment group between our in vitro and in vivo models and the abrogation of pre-treatment protection in our in vitro model. Future studies should consider using primary proximal tubular epithelial cells to assess if such mRNA and protein differences influence cell death outcomes in an in vitro model. Additionally, the use of 37 °C hypoxia in our in vitro model may also partially explain the discrepancy in protection found in our combination group between our in vitro and in vivo models since ischemia occurred at 4 °C in our in vivo model. The use of 37 °C hypoxia in our in vitro model in the present study may also explain the discrepancy in protection found between the STS pre-treatment combined with the hypoxic treatment group and its corresponding group in our in vivo model, where ischemia occurred at 4 °C. However, the finding that STS pre-treatment alone is protective when administered at 37 °C is particularly important since, in the context of clinical transplantation, the body temperature of a living kidney donor would be normothermic.

In our in vivo rat model of syngeneic kidney transplantation, STS pre-treatment combined with UW+STS had the highest survival rate of all the experimental groups, but this survival was not significantly different compared to control (Saline Pre/UW). The lack of a pairwise significant difference (Holm–Bonferroni procedure) despite a global significant difference (log-rank test) is a phenomenon that can occur and may have been due to the high number of comparisons combined with the low sample sizes of some groups [39,40]. In contrast, both monotherapy treatments (STS pre-treatment alone and UW+STS alone) had relatively smaller impacts on transplant recipient survival compared to the control, with no recipients in the UW+STS alone group surviving to POD-3. These data suggest that combining STS pre-treatment with supplementation to UW solution may improve survival following transplantation. However, we found that none of the treatments led to significant improvements in the blood markers of renal function assessed in this study, although pre-treatment alone and combination treatment showed a downward trend in terminal serum creatine. The lack of an effect on terminal renal function may be due to high variability as a result of temporal changes in blood marker levels post-transplantation, which has been shown in human, porcine, and rat kidney transplantation [41,42,43]. The limited number of recipient rats that survived to POD-3 in this study, with only one recipient rat in the control group surviving to POD-3, may explain the lack of an effect of pre-treatment on renal function at this time point. Other studies have shown that H_2_S pre-treatment in models of warm renal IRI reduced plasma creatinine levels following reperfusion [44,45,46]. Moreover, previous studies using the same rat model of kidney transplantation have shown that renal function and recipient survival are closely linked [29,47,48]. Considering that the combination treatment group had the best survival rate of the experimental groups, combined with the decreasing trend observed in terminal serum creatinine, STS pre-treatment combined with supplementation to preservation solution may improve renal function.

We also found that none of the treatments significantly affected urine osmolality and production, with urine osmolality being lower and urine production higher in all the experimental groups compared to Sham. In the context of clinical kidney transplantation, immediate production of urine is an important indicator of graft function and is significantly associated with reduced occurrence of delayed graft function along with improved graft and patient survival [49,50]. A previous study using the same in vivo model found that differences in recipient urine osmolality and volume between experimental groups widened as post-transplantation time increased from POD-3 to POD-14, which may explain the minimal differences observed on POD-3 in this study [29].

We found no significant differences in terminal and POD-3 oxidative DNA damage in any of the treatment groups compared to the control, as assessed through 8-OHdG staining. Our assessment of lipid peroxidation (an indicator of oxidative damage) in the form of 4-HNE revealed no significant differences in terminal and POD-3 lipid peroxidation levels. However, we found a decreasing trend in lipid peroxidation levels in the STS pre-treatment and combination treatment groups for both our terminal and POD-3 analyses. Other studies have also found that treatment with NaHS reduces 4-HNE levels in the context of ureteral obstruction in kidneys and neuronal cells exposed to formaldehyde [51,52]. A possible mechanism through which STS may inhibit lipid peroxidation is via the Nrf2 pathway. Yang et al. showed that H_2_S persulfidates Keap1, which causes Keap1 to dissociate from Nrf2, thereby enabling Nrf2 to translocate from the cytoplasm to the nucleus where it upregulates the expression of antioxidant genes [53]. One such protein that is upregulated upon Nrf2 nuclear translocation is GPX4, an enzyme responsible for the conversion of toxic lipid hydroperoxides generated from ROS to non-harmful derivatives [54,55]. Notably, of the three antioxidant genes we examined in this study, terminal and POD-3 GPX4 mRNA expression showed a consistent non-significant upward trend in both the STS pre-treatment and combination treatment groups relative to control, reaching Sham levels. Interestingly, Calvert et al. found that intravenous injection of sodium sulfide, an H_2_S donor, in mice significantly increased nuclear levels of Nrf2 within 30 min of injection, which remained elevated for two hours [56]. Similarly, we intravenously injected the donor rats in our study with STS 30 min before their kidneys were procured. These findings suggest that STS pre-treatment may increase nuclear Nrf2 levels in the kidneys before procurement, leading to the upregulation of various antioxidant genes, such as GPX4 before ischemia, thereby providing proactive protection against IRI. Future studies should assess whether STS pre-treatment does indeed increase nuclear Nrf2 translocation to confirm this proposed mechanism.

The possibility that STS pre-treatment upregulated GPX4 expression and suppressed lipid peroxidation may also explain our ATN findings in the present study. We found downward trends in terminal ATN scores for the STS pre-treatment and combination treatment groups, which became significant in POD-3 grafts only. GPX4 is a key regulator of ferroptosis, a form of regulated necrosis that is partly caused by the accumulation of high levels of lipid peroxides, which GPX4 reduces [55,57]. Indeed, reduced GPX4 expression and increased levels of 4-HNE are often used as markers of ferroptosis [58,59,60]. Ferroptosis has also been suggested as a major form of necrosis in renal IRI [19]. Since STS pre-treatment and combination treatment showed upward trends in GPX4 expression and downward trends in 4-HNE levels, the major form of necrosis inhibited by STS pre-treatment may be ferroptosis. This proposed mechanism is further corroborated by Zhang et al. in a recent study that showed NaHS treatment of cardiomyocytes inhibits ferroptosis by activating Nrf2 signaling through persulfidation of Keap1 [54].

We also assessed tissue levels of p-MLKL, a marker of necroptosis, which is another form of regulated necrosis suggested to play a major role in renal IRI along with ferroptosis [19,61,62]. Unexpectedly, we found no significant differences in p-MLKL levels. Although we observed downward trends in terminal p-MLKL levels in the pre-treatment and combination treatment groups, these trends were absent on POD-3. Since STS pre-treatment and combination treatment showed downward trends in terminal p-MLKL levels, which included recipient rats from earlier time points, it is possible that STS pre-treatment may primarily influence necroptosis at earlier stages of reperfusion. It is also possible that STS pre-treatment suppressed the recently discovered RIPK3-Ca^2+^/calmodulin-dependent protein kinase II (CaMKII) pathway of necroptosis, which may be influenced by H_2_S and does not involve MLKL [63,64,65]. Accordingly, future studies should examine the effect of STS pre-treatment on necroptosis at earlier stages of reperfusion and the RIPK3-CaMKII pathway of necroptosis. 

Although necrosis and regulated forms of necrosis are key contributors to renal IRI, apoptosis also plays a contributing role. We found, through TUNEL staining, that both STS pre-treatment and combination treatment showed decreasing trends in terminal tissue apoptosis levels compared to control, with these differences becoming significant on POD-3. Apoptosis was reduced by at least 20% in both groups at both time points, suggesting that inhibition of apoptosis is a major mechanism of protection conferred by STS against renal IRI. As previously discussed, thiosulfate can bind caspase 3 and persulfidate a cysteine residue, thereby inactivating caspase 3 [34,35]. However, another study showed that Nrf2 can upregulate the expression of BCL-2, an anti-apoptotic protein [66]. In that study, upregulation of BCL-2 reduced pro-apoptotic BAX protein expression, mitochondrial cytochrome *c* release, and caspase 3/7 activity, collectively inhibiting apoptosis [66]. Our finding that STS pre-treatment and combination treatment had upward trends in GPX4 expression, which is also activated by Nrf2, further supports this mechanism of action for inhibiting apoptosis. Thus, STS may have also protected against apoptosis by activation of Nrf2, which confirms the anti-apoptotic effects of STS and other H_2_S donors in our present and previous studies [29,47,48,67,68].

In addition to cell death, we also found upward trends in overall macrophage infiltration in all experimental groups relative to Sham at both time points. This finding was initially unexpected due to the known anti-inflammatory effects of STS and other H_2_S donors [29,47,67,68]. However, compared to the control group, the non-significant increases in macrophage infiltration were marginal for both the STS pre-treatment and combination treatment groups, with these differences being less than 1%. Previous research has shown that renal tissue levels of macrophages progressively increase from POD-0 to POD-7 following renal IRI [69]. Since both STS pre-treatment and combination treatment had higher trends in POD-3 survival compared to control, this extended survival time may have enabled these temporal-based increases in macrophage infiltration to occur following IRI. We also found upward trends in terminal and POD-3 M2 macrophages in both pre-treatment groups relative to the control and Sham groups, with terminal differences compared to the Sham group being significant. In the context of renal IRI, pro-inflammatory M1 macrophages have been shown to exacerbate damage to renal tubules following IRI, whereas M2 macrophages are protective [69,70]. Although it is possible that both M1 and M2 macrophage populations increased proportionally in the STS pre-treatment and combination treatment groups, other studies have shown that H_2_S can induce macrophage polarization to an M2 state [71,72]. Thus, STS pre-treatment may promote macrophage polarization to an M2 state which in turn may have promoted renal tubule repair and inhibited pathological inflammation. However, further research investigating the effect of STS on macrophage polarization in the context of transplantation is needed to establish this effect. Our finding that STS pre-treatment and combination treatment reduced terminal neutrophil infiltration also supports STS promoting an anti-inflammatory environment following IRI. The combination treatment also reduced neutrophil infiltration on POD-3 while pre-treatment alone showed a similar but non-significant decrease. Previous studies from our group have shown that STS and other H_2_S donors suppress neutrophil infiltration following renal IRI [29,47,68]. The underlying mechanism through which these H_2_S donors reduce neutrophil infiltration may be due to impaired adherence and extravasation of neutrophils from the circulation across the endothelium into the tissue [67,73].

**Figure 10:**
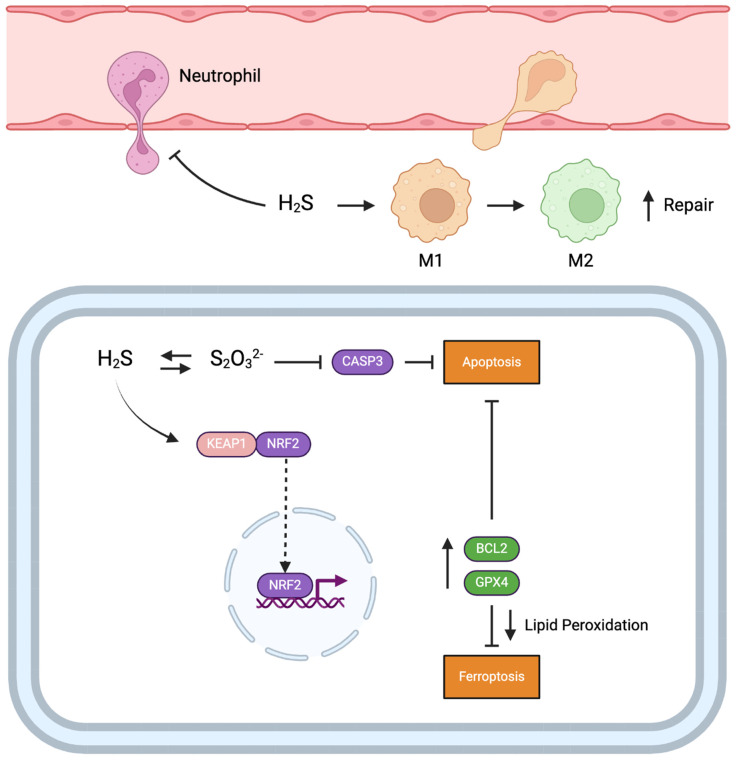
Proposed mechanisms of protection mediated by STS pre-treatment. Thiosulfate is converted to H_2_S and vice-versa. We propose that H_2_S produced from thiosulfate persulfidates KEAP1, enabling NRF2 nuclear translocation and subsequent upregulation of BCL2 and GPX4. BCL2 inhibits apoptosis while GPX4 inhibits ferroptosis by reducing lipid peroxidation. Thiosulfate also inhibits apoptosis through persulfidation of CASP3. H_2_S inhibits neutrophil adherence to the endothelium and subsequent extravasation while promoting M2 macrophage polarization. H_2_S: hydrogen sulfide; S_2_O_3_^2−^: thiosulfate; CASP3: caspase 3; KEAP1: Kelch-like ECH-associated protein 1; NRF2: Nuclear factor erythroid 2-related factor 2; BCL2: B-cell lymphoma 2; GPX4: glutathione peroxidase 4; M1: M1 macrophage; M2: M2 macrophage; STS: sodium thiosulfate. Figure created with BioRender.com.

Finally, we found that STS pre-treatment and combination treatment showed downward trends in terminal and POD-3 tissue KIM-1 levels. KIM-1 is a membrane protein normally expressed at low levels in renal tubular epithelial cells of normal healthy kidneys [74]. However, KIM-1 expression is upregulated following ischemia, particularly in epithelial cells found in damaged parts of the kidney, especially the proximal tubule [74]. Urinary KIM-1 levels are also associated with the severity of acute renal failure and show prognostic value in predicting outcomes for patients with acute renal failure [75]. Since KIM-1 is a marker of tubular injury, the downward trend in KIM-1 expression in both the STS pre-treatment and combination treatment groups aligns with the improvements in ATN, apoptosis, and neutrophil inflammation observed in the present study. A schematic illustration summarizing all the proposed mechanisms of STS pre-treatment discussed in this study can be found below (Figure 10).

Although useful for preliminary studies, our in vitro model of hypoxia–reoxygenation injury induced hypoxia at 37 °C while kidneys were ischemic at 4 °C in our in vivo model of kidney transplantation. The reason for this temperature difference was due to the technological inability of our hypoxia chamber to maintain hypoxia at 4 °C. As previously mentioned, this discrepancy in temperature may explain the different outcomes observed in our combination treatment group between the two models. A potential solution to this challenge is to use anaerobic sachets that are placed in a fridge at 4 °C. However, the method of hypoxia induced by these anaerobic sachets differs from the method used by our hypoxia chamber and does not allow precise control over oxygen concentrations. Hypoxia chambers are also commonly used in other studies that rely on in vitro models of hypoxia–reoxygenation injury [46,76,77,78]. We also decided to pre-treat cells with an STS concentration of 150 μM based on a previous study that showed that this concentration was most protective when administered during hypoxia, but not before hypoxia [29]. The most protective STS concentration for pre-treatment of cells may differ from the concentration used for hypoxic treatment. As a result, future research should focus on conducting a dose-response study for STS pre-treatment specifically. This information could also help determine the optimal dosage for use in our in vivo model.

In our in vivo model, kidneys were subjected to SCS for 24 h, which represents an extended period of cold ischemia normally associated with transport of the kidney between different transplant centers, rather than within the same center [79,80]. Thus, the model used in the present study allowed us to determine whether STS pre-treatment could protect against the more severe forms of IRI observed in the clinic. However, this model of extreme cold storage elicited a high amount of damage, which may have negatively impacted recipient rat survival. This variability in survival hindered the ability to make accurate comparisons due to temporal changes in IRI severity post-transplantation [41,42,43,69]. As a result, we performed subgroup analysis to show POD-3 exclusive outcomes, but the sample sizes of the groups at this time point were small, reducing the statistical power of our study. A possible solution could be to shorten the SCS time to reduce damage, but this could potentially reduce the treatment effect observed. Another possibility could be to use the same length of SCS but preemptively sacrifice recipient rats at an earlier time post-transplantation. Another limitation of our study is that we assessed survival to POD-3 rather than long-term survival. However, a previous study using this model of kidney transplantation found that survival to POD-14 after POD-3 remained relatively unchanged for all groups [29].

We also used a syngeneic model to eliminate the confounding variable of graft rejection. Although this choice helped us narrow our focus to inflammation and injury caused by IRI only, syngeneic kidney transplantation only applies to kidney transplants between identical twins. Most kidney transplants performed are allogeneic, where the genetic backgrounds of the recipient and donor are different. The literature surrounding the effects of H_2_S on alloimmune responses and graft rejection in the context of allogeneic kidney transplantation is relatively scarce. Two previous studies in our lab found that supplementation of NaHS to UW solution protected against renal IRI in allogeneic rat models of kidney transplantation but not graft rejection [48,81]. However, these studies focused on supplementation of an H_2_S donor to preservation solution during ischemia and used a different H_2_S donor, NaHS. Future studies should therefore examine STS pre-treatment in the context of allogeneic kidney transplantation but also assess the effect of H_2_S on alloimmune responses more broadly considering the lack of research in this area. Indeed, our syngeneic model suggests that STS pre-treatment inhibits neutrophil inflammation and may induce macrophage polarization to an M2 state, which could be beneficial in the context of allogeneic transplantation. As previously mentioned, the dose of STS used for pre-treatment should be further investigated via a dose–response study, which could be performed using either our in vitro or in vivo models. Another important consideration is the timing of STS administration. We pre-treated donor rats in our in vivo model with STS 30 min before kidney procurement based on the reported plasma half-life of STS being 26 min in guinea pigs [82]. It is possible that the half-life of STS in rats may differ which could influence the optimal pre-treatment time.

Additionally, it is unclear whether the 30 min pre-treatment time and 150 μM concentration of STS used in this study were sufficient to pre-condition the donor kidneys before removal. It is possible that no protective pathways were upregulated prior to donor kidney removal and instead the STS injected into the donor largely mediated protection during cold storage. However, Marutani et al. found that incubation of activated caspase 3 with 250 μM STS for 30 min reduced caspase 3 activity, and incubation of neuroblastoma cell lysates with STS using the same amount of time and concentration resulted in the persulfidation of caspase 3 [34]. These findings suggest that 30 min may be sufficient time for STS to mediate protection, at least through caspase 3 inhibition. Thus, STS pre-treatment for 30 min may have helped pre-condition donor kidneys against apoptosis prior to removal. However, these findings used a higher STS concentration in an in vitro context, which does not account for the elimination half-life of STS and the time required for thiosulfate to circulate in the bloodstream, penetrate tissue, and enter renal cells following intravenous injection. In the same study by Marutani et al., the researchers found that intracellular thiosulfate levels were significantly elevated in neuroblastoma cells in vitro following three hours of incubation with 250 μM STS, but not after one minute [34]. Additionally, the researchers found that thiosulfate levels were significantly elevated in the brain tissue of mice 90 min following intraperitoneal injection of 10 mg/kg STS [34]. Thus, the collective amount of time required for STS to circulate, penetrate tissue, enter cells, and upregulate protective pathways following administration at a specific concentration is unclear. To our knowledge, no studies have investigated the concentration and treatment time required for STS to activate protective pathways in kidneys in an in vivo context. Future studies should therefore investigate the concentration and time required for intravenously injected STS to activate protective pathways prior to donor kidney removal, ensuring pre-conditioning of the kidney before removal as opposed to direct treatment during cold storage. Finally, our in vivo model reflected living donor kidney transplantation but not donation-after-circulatory-death (DCD) or donation-after-brain-death (DBD) transplantation. STS pre-treatment would currently be most applicable in the context of living donors and DBD kidney transplantation since donor circulation would need to be active for injected STS to reach the kidney. As a result, future studies should examine the effect of STS in models of transplantation involving DBD donors. However, with the advent of abdominal normothermic regional perfusion, where the kidney is perfused in situ with oxygenated blood following death, STS pre-treatment could be used for DCD donors as this technology expands [83,84].

A surprising finding in our study was that the UW+STS alone treatment group had the worst survival of all the experimental groups, which was associated with poor renal function and histological outcomes. In our previous study that used the same rat model of syngeneic kidney transplantation, STS supplementation to UW solution improved survival, renal function, and renal graft histology [29]. This discrepancy may be due to the total sample size of the UW+STS alone group being far smaller than the total sample sizes of the other experimental groups in this study and all groups in the previous study. As well, our current study was required to employ a standardized scoring algorithm for monitoring the welfare of the recipient rats and deciding when to preemptively sacrifice, whereas the previous study did not use a scoring algorithm. The stringent scoring algorithm used in the current study could have increased the likelihood a recipient rat was preemptively sacrificed compared to the previous study, thereby reducing overall survival. This difference in animal monitoring, along with the low sample size, makes it difficult to accurately compare the UW+STS alone group in the present study to the previous study.

## 4. Methods

### 4.1. In Vitro Model of Rat Renal Epithelial Cell Hypoxia–Reoxygenation Injury

A previously established in vitro model of rat renal epithelial cell hypoxia–reoxygenation injury was used to assess whether STS pre-treatment could protect against hypoxia–reoxygenation injury (Supplementary Figure S1) [29]. Since renal proximal tubular epithelial cells are particularly susceptible to IRI, rat kidney proximal tubular epithelial cells (NRK-52E, ATCC, USA) were used, which is also consistent with our in vivo rat model of kidney transplantation [85]. Cells were incubated under normal growth conditions of 37 °C, 5% CO_2_, and 21% O_2_ in Dulbecco’s modified eagle medium (DMEM) supplemented with 10% fetal bovine serum (FBS) and 1% penicillin/streptomycin (P/S). Cells were passaged and seeded into 6-well plates overnight for experiments using 0.25% trypsin–EDTA. Normoxia control cells were cultured under the same conditions as pre-experimental cells. Experimental cells were incubated in hypoxic conditions (5% CO_2_, 0.5% O_2_, 95% N_2_) for 24 h at 37 °C in serum-free DMEM using the HypOxstation H85 hypoxia chamber (HYPO_2_YGEN, Frederick, MD, USA) to simulate ischemia. After hypoxia, cells were incubated under normal growth conditions (37 °C, 5% CO_2_, 21% O_2_) in serum-positive DMEM for 24 h to simulate reperfusion. Experimental cells were treated with 150 μM of STS two hours before hypoxia and/or during hypoxia, while control (untreated) cells received no STS treatment. STS was obtained from a 250 mg/mL injectable solution of STS (Seaford Pharmaceuticals Inc., Mississauga, ON, Canada). Following reoxygenation, both control and experimental cells were stained with FITC-conjugated Annexin V (BioLegend, San Diego, CA, USA) and propidium iodide (BioLegend, San Diego, CA, USA) to assess apoptosis and necrosis, respectively. Cellular staining was analyzed via flow cytometry using CytoFLEX S (Beckman Coulter, Indianapolis, IN, USA). CytExpert Software 2.5 (Beckman Coulter, Indianapolis, IN, USA) was used to appropriately gate the data for statistical analysis. Compensation was performed using unstained live cells and a mix of live and heat-killed cells stained with either FITC-conjugated Annexin V or propidium iodide.

### 4.2. Experimental Animals

A previously established in vivo rat model of syngeneic kidney transplantation was used to assess whether STS pre-treatment could protect against transplantation-induced IRI (Supplementary Figure S2) [29]. Male Lewis rats weighing 250–385 g were purchased from Charles River Canada (St. Constant, QC, Canada). Rats were housed in the Animal Care and Veterinary Services (ACVS) facility at Western University (London, ON, Canada) prior to surgery under standard conditions. All animal studies were approved by the Western University Council on Animal Care and Animal Use under animal-use protocol number 2023-019. Rats were allocated into five different groups: Sham (n = 3), Saline Pre/UW (n = 5), STS Pre/UW (n = 6), Saline Pre/UW+STS (n = 3), and STS Pre/UW+STS (n = 7).

### 4.3. Kidney Donor Pre-Treatment

Donor rats were anesthetized via an intraperitoneal injection of ketamine/xylazine and placed on a warm water circulating heat pad set to 37 °C to maintain body temperature continuously throughout donor pre-treatment and renal graft procurement. After being anesthetized, donor rats received a lateral tail vein injection of 0.2 mL of 37 °C pre-warmed PBS (Saline Pre/UW, Saline Pre/UW+STS) or PBS + 2.4 mg STS/kg body weight (STS Pre/UW, STS Pre/UW+STS) 30 min before kidney procurement using a 25-G needle. The 2.4 mg STS/kg body weight dose was chosen since this was estimated to establish a blood concentration of 150 μM STS. The 30 min injection timepoint was used to account for the half-life of STS in rodents [82]. Following tail vein injection, donor rats were transferred to the microsurgeon for removal of the donor kidney.

### 4.4. Syngeneic Kidney Transplantation and Animal Monitoring

Syngeneic rat kidney transplantation was performed in Lewis rats to eliminate any possible confounding effects of immunosuppression. Following intraperitoneal anesthetic induction with ketamine/xylazine, rats were maintained under general anesthesia with isoflurane during surgery. Using aseptic techniques, the left donor kidneys were perfused with heparinized Ringer’s lactate solution and flushed with 10 mL of 4 °C UW preservation solution (Saline Pre/UW, STS Pre/UW) or UW + 150 μM STS (STS Pre/UW+STS, Saline Pre/UW+STS) until venous effluent was clear. After flushing, kidney grafts were procured and stored in 50 mL of the same perfusion solution in a 50 mL sterile conical tube at 4 °C for 24 h. This cold storage time was previously shown to result in graft function loss in untreated animals [29]. Recipient rats underwent bilateral nephrectomy followed by renal transplantation via end-to-side anastomosis of the donor kidney vascular supply to the recipient aorta and inferior vena cava with 11-0 or 10-0 nylon sutures. Excess connective tissue on the kidney graft was fastened to the retroperitoneal space of the recipient using nylon sutures. Urethral reconstruction was performed by anastomosis of the donor ureter and ureterovesical junction to the recipient’s bladder using interrupted 11-0 monofilament nylon sutures. A bilateral nephrectomy of the recipient’s native kidneys was performed to ensure the recipient’s renal function solely depended on the transplanted kidney graft. Sham rats received a midline incision only. All surgeries were under 3 h and performed by the same microsurgeon, who was blinded to the experimental design. Graft failure was presumed in animals that died or required premature sacrifice based on a standardized animal scoring algorithm. All rats were monitored until post-operative day (POD) 3 or until premature sacrifice. The post-operative period of 3 days was chosen since it was previously shown that differences in outcomes in this model emerge by this time point and most untreated rats do not survive past this day [29].

### 4.5. Serum Creatinine, BUN, and eGFR

Blood samples were collected from transplant recipient and Sham-operated rats upon sacrifice via intracardiac puncture and centrifuged at 2000× *g* for 15 min at 4 °C to obtain serum. The supernatant (serum) was aspirated and stored at −80 °C until analysis. Serum creatinine and BUN levels were determined using an IDEXX Catalyst One Chemistry Analyzer (Markham, ON, Canada). The estimated glomerular filtration rate (eGFR) was calculated using rat mass in grams (W), serum creatinine (C), and BUN (U) via Equations (1) and (2) [86].

Serum creatinine < 52 μM: eGFR = 880 × W^0.695^ × C^−0.660^ × U^−0.391^ (1)

Serum creatinine ≥ 52 μM: eGFR = 5862 × W^0.695^ × C^−1.150^ × U^−0.391^ (2)

### 4.6. Urine Osmolality

Transplant recipient and Sham-operated rats were placed in metabolic cages for 24 h from POD-2 to POD-3 for urine collection. Total urine volume was recorded and urine samples were stored at −20 °C for urine osmolality analysis. Urine osmolality was measured via freezing-point osmometry using the 3320 Osmometer (Advanced Instruments, Norwood, MA, USA) and compared to company-provided standards.

### 4.7. Renal Tissue Processing

Renal grafts were obtained from recipients and Sham-operated rats upon sacrifice. Grafts were then weighed and frontally halved. One half was placed in 10% formalin for a minimum of 72 h and a maximum of 1 week followed by storage in 70% ethanol for later paraffin embedding and sectioning. The other half was placed in RNA*later* solution (Thermo Fisher Scientific, Waltham, MA, USA) to inhibit RNA degradation for later quantitative PCR (qPCR) analysis. Samples were stored in RNA*later* at 4 °C for a minimum of 24 h and a maximum of 1 week to ensure the solution thoroughly penetrated the tissue and then stored at −80 °C for later analysis.

### 4.8. Histological Staining

Formalin-fixed kidneys were embedded with paraffin, cut into 5 μm thick sections, and placed onto microscopic slides for staining. Paraffinization, sectioning, and staining were all performed in a blinded fashion. Sections were stained with Hematoxylin and Eosin (HE) and Terminal deoxynucleotidyl transferase dUTP nick end labeling (TUNEL) to determine the degree of ATN and apoptosis, respectively. H&E sections were scored for ATN by a blinded renal pathologist using the following scheme: 1 = < 11%, 2 = 11–24%, 3 = 25–45%, 4 = 46–75%, 5 = > 75% graft ATN [29]. 

Kidney sections also underwent immunohistochemical staining with primary antibodies against Kidney Injury Molecule-1 (KIM-1) (Cat No: AF3689, R&D Systems, Minneapolis, MN, USA), neutrophil marker myeloperoxidase (MPO) (Cat No: ab9535, Abcam, Cambridge, UK), pan-macrophage marker CD68 (Cat No: ab955, Abcam), M2 macrophage marker CD163 (Cat No: ab182422, Abcam), necroptosis marker phospho-mixed lineage kinase domain-like (p-MLKL) (Cat No: PA5-105678, Thermo Fisher Scientific), lipid peroxidation marker 4-Hydroxynonenal (4-HNE) (Cat No: ab48506, Abcam), and oxidative DNA damage marker 8-hydroxy-2′-deoxyguanosine (8-OHdG) (Cat No: ab48508, Abcam). Primary antibody staining was visualized via secondary antibodies and 3,3′-Diaminobenzidine (DAB) chromogen substrate using the Dako Envision System (Dako, Glostrup, Denmark) as per the manufacturer’s protocol.

### 4.9. Whole-Slide Image Analysis

Stained sections were scanned at 40× magnification using a Leica Aperio AT2 scanner (Leica Biosystems Inc., Concord, ON, Canada) to generate whole-slide images. Sections that underwent TUNEL and immunohistochemical staining were analyzed using QuPath Version 0.5.1 [87]. The wand tool was used to identify and annotate the whole kidney section and the pixel classification tool was used to calculate the percentage of total positively stained area (brown) of the whole kidney section for each stain [88]. The pixel threshold was adjusted for each stain and the same threshold was applied across all sections for each stain.

### 4.10. Quantitative PCR and Analysis

Renal grafts stored at −80 °C in RNA*later* solution were thawed and ~30 mg samples of renal tissue were excised from the renal cortex. The excised renal tissues were homogenized using a rotor–stator homogenizer and total RNA was isolated using RNeasy Mini Kit (Qiagen, Toronto, ON, Canada). Total RNA concentration and purity were measured using a GENESYS 10S UV-Vis spectrophotometer (Thermo Fisher Scientific) and reverse transcribed into cDNA using OneScript Plus cDNA Synthesis Kit (Applied Biological Materials, Vancouver, BC, Canada) with Oligo(dT) primers according to kit manufacturer instructions. qPCR reactions were prepared using the BlasTaq 2X qPCR Master Mix (Applied Biological Materials, Vancouver, BC, Canada) according to the kit manufacturer’s instructions to a final volume of 20 µL per reaction. For each gene tested, reactions were run in triplicate with a no template control on a 96-well plate. qPCR was performed using QuantStudio 3 Real-Time PCR System (Thermo Fisher Scientific). Primer sequences were designed using Primer-BLAST (NCBI) that targeted catalase and glutathione peroxidase 4 (GPX4). Primer sequences for Glyceraldehyde 3-phosphate dehydrogenase (GAPDH) and superoxide dismutase 1 (SOD-1) were designed as previously described [89,90]. Primer sequences are shown in Supplementary Table S1. All target genes were normalized against GAPDH gene expression and fold changes of gene expression relative to Sham-operated rats were calculated using the delta delta (∆∆)Ct method.

### 4.11. Statistical Analysis

All statistical analyses and graphs were generated using GraphPad Prism Version 10.2.0. POD-3 survival data were analyzed using Kaplan–Meier survival analysis and log-rank test. Post-hoc analysis of survival curves was performed via the Holm–Bonferroni method using the survival and survminer packages in RStudio version 4.3.3. All other data were analyzed using one-way ANOVA and Tukey’s post-hoc tests to assess statistical differences. Data are represented as mean ± SEM unless otherwise stated. Statistical significance was accepted at *p* < 0.05.

## 5. Conclusions

In conclusion, our study provides evidence that STS pre-treatment may protect against renal IRI induced by kidney transplantation, which may lead to an overall improvement in transplant recipient survival. The potential underlying mechanisms of STS pre-treatment identified included protection against apoptosis, ATN, and neutrophil inflammation. This protection may be attributed to reduced oxidative stress and ferroptosis, but future studies must confirm this link. To our knowledge, this is the first experimental study showing that STS pre-treatment confers organ protection in the context of kidney transplantation. Our results suggest that STS pre-treatment could be a clinically viable and inexpensive method for protecting against IRI and improving transplantation outcomes for transplant recipients. Our study helps provide a rationale for conducting expanded STS pre-treatment studies in rats and possibly larger animal models, such as pig models.

## Figures and Tables

**Table 1:** Description of animal groups used for in vivo model of rat kidney transplantation.

Animal Group	Description **
Sham *	Midline incision only
Saline Pre/UW (control)	PBS pre-treatment, graft storage in UW solution
STS Pre/UW	STS pre-treatment, graft storage in UW solution
Saline Pre/UW+STS	PBS pre-treatment, graft storage in UW+STS solution
STS Pre/UW+STS	STS pre-treatment, graft storage in UW+STS solution

* Sham rats did not receive a kidney transplant. ** All pre-treatment injections were performed intravenously 30 min before donor kidney procurement and procured renal grafts were stored on ice at 4 °C for 24 h followed by transplantation into bilaterally nephrectomized recipient rats.

## Data Availability

The original contributions presented in this study are included in the article.

## Supplementary Materials

The following supporting information can be downloaded at: https://www.mdpi.com/article/10.3390/ijms25179529/s1.
