# Supplementary material for: Effect of Sodium Thiosulfate Pre-Treatment on Renal Ischemia-Reperfusion Injury in Kidney Transplantation
Source: Int J Mol Sci. 2024 Sep 2;25(17):9529. doi: 10.3390/ijms25179529 (PMC11395123; doi:10.3390/ijms25179529)
Supplement: Supplementary file 1 [file ijms-25-09529-s001.zip › ijms-3174374-supplementary.pdf]

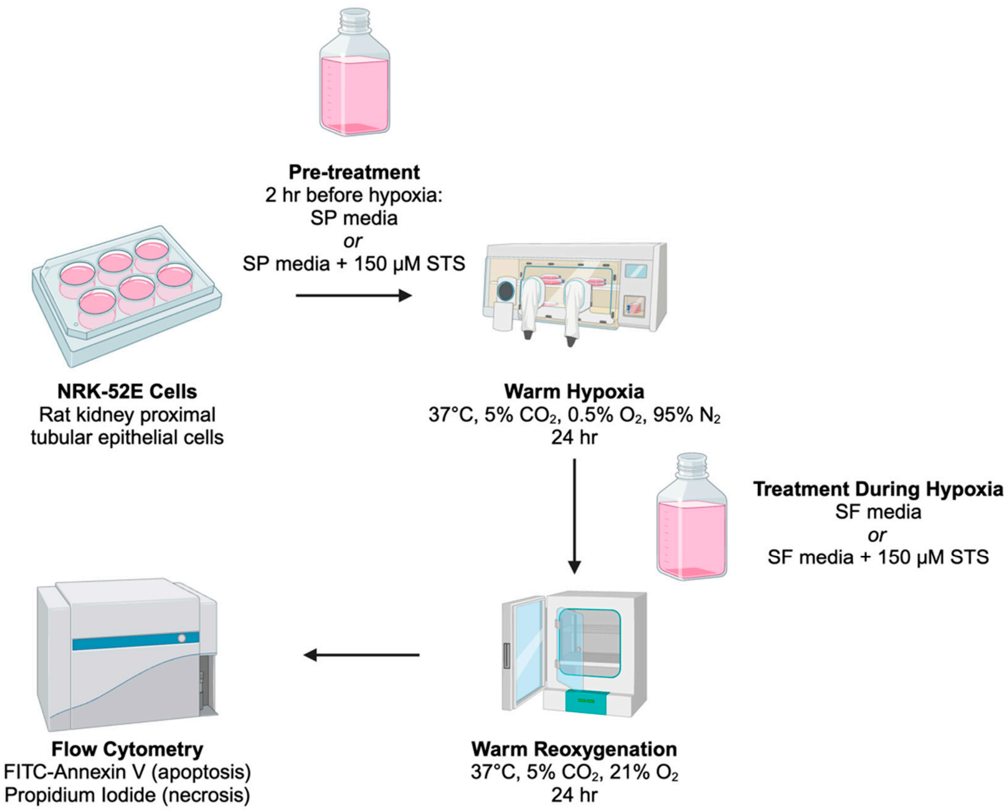

Supplementary Figure S1. *In vitro* model of rat renal epithelial cell hypoxia-reoxygenation injury. Rat proximal tubular epithelial cells (NRK-52E) were pre-treated with 150 µM STS 2 hours before and/or during 24 hours of warm hypoxia. Cells were reoxygenated for 24 hours, stained with FITC-Annexin V and propidium iodide, and analysed via flow cytometry. SP: serum-positive; SF: serum-negative. Figure created with BioRender.com.

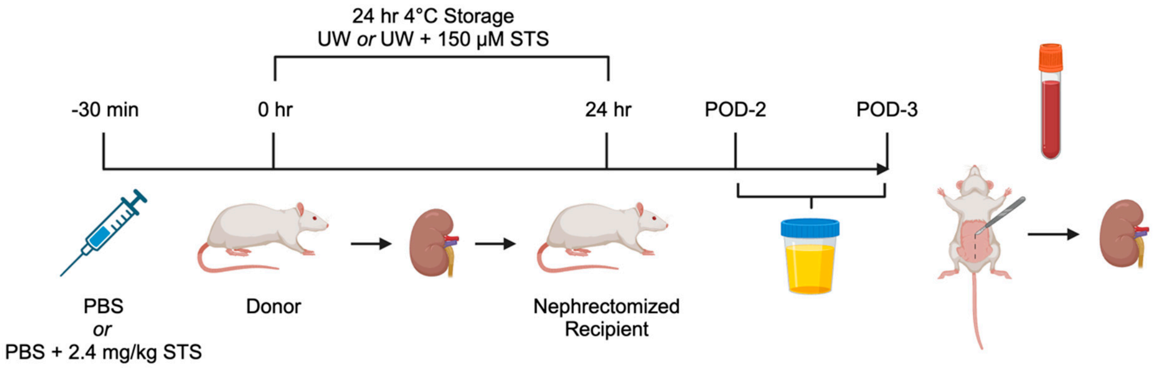

Supplementary Figure S2. *In vivo* rat model of syngeneic kidney transplantation. Donor rats received an intravenous injection of PBS or PBS + 2.4 mg/kg STS 30 minutes before kidney procurement. Renal grafts were stored in UW or UW+150 µM STS for 24 hours at 4°C and transplanted into bilaterally nephrectomised recipient rats. Urine was collected from POD-2 to POD-3, at which point recipient rats were sacrificed to obtain blood samples and the renal grafts. POD: post-operative day; PBS: phosphate-buffered saline. Figure created with BioRender.com.

Supplementary Table S1. Primer sequences used for qPCR of rat renal tissue.

Primer Name	Forward Sequence (5'–3')	Reverse Sequence (5'–3')
GAPDH	GACATGCCGCCTGGAGAAAC	AGCCCAGGATGCCCTTTAGT
Catalase	CAGGGATGCCATGTTGTTTCC	GCCATTCATGTGCCGATGTC
SOD-1	TAGCAGGACAGCAGATGAGT	GCAGAAGGCAAGCGGTGAAC
GPX4	CCGTCTGAGCCGCTTATTGA	CACACGCAACCCCTGTACTT
